# Dedifferentiated chondrsarcoma: a clinicopathologic analysis of 25 cases

**DOI:** 10.1186/s12891-021-04053-7

**Published:** 2021-02-15

**Authors:** Lei Cao, Yuan Wu, Shu-Man Han, Tao Sun, Bao-Hai Yu, Feng Gao, Wen-Juan Wu, Bu-Lang Gao

**Affiliations:** 1 Department of Radiology, The Third Hospital of Hebei Medical University, 139 Ziqiang Road, Shijiazhuang, 050051 Hebei Province China; 2 Hebei Provincial Gucheng County Hospital, Gucheng, 253800 Hebei Province China

**Keywords:** Dedifferentiated chondrosarcoma, Imaging, Microscopy, Pathology, Diagnosis

## Abstract

**Background:**

To investigate the clinical, imaging and pathological features of dedifferentiated chondrosarcoma for better diagnosis.

**Methods:**

Patients who had been confirmed to have dedifferentiated chondrosarcoma were enrolled in this study and analyzed in the clinical, imaging and pathological data.

**Results:**

Twenty-five patients had pathologically confirmed dedifferentiated chondrosarcoma including 15 males and 10 females with an age range of 24–74 (median 58, interquartile range 49–65). Ten patients had the tumor at the femur, four at the ilium, two at the humerus, two at the tibia, two at cotyle, and one at each of the following locations: scapula, sacrum, rib, pubic branch, and calcaneus. Twenty-one patients had local pain and a soft tissue mass while the other four patients had only local pain without a soft tissue mass. Four patients had pathological fractures. Imaging showed extensive bone destruction with calcification inside the lesion and possible pathological fractures. On gross observation of the specimen, the chondrosarcoma components were usually located inside the bone, and the dedifferentiated sarcoma components were mainly located outside the bone. Microscopy showed the dedifferentiated tumor had two components: well-differentiated chondrosarcoma and poorly differentiated non-chondral sarcoma including malignant fibrous histiocytoma in eleven cases, osteosarcoma in ten cases, fibrosarcoma in two, liomyosarcoma in one, and lipoblastoma in the remaining one.. Followed up from 3 moths to 60 months (mean 15.6), eight patients died with a survival time of 10–23 months (mean 16), and the other 17 patients survived with the survival duration from three to 60 months (15).

**Conclusion:**

Dedifferentiated chondrosarcoma is a fatal disease with multiple components, and most of the cases have dual morphological and imaging features of chondrosarcoma and non-chondrosarcoma. The imaging presentations are primarily of common central chondrosarcoma, combined with cortical destruction, soft tissue mass, and pathological fractures.

## Background

As a rare, highly malignant form of chondrosarcoma firstly described by Dahlin and Bebout in 1971 [1], dedifferentiated chondrosarcoma accounts for up to 11% of all chondrosarcoma cases and is characterized by well-differentiated cartilaginous component of low-grade chondrosarcoma adjacent to dedifferentiated malignant mesenchymal tumor of high-malignancy grade with an abrupt interface between these two components [2–9]. The dedifferentiated component may have various characteristics like osteosarcoma, angiosarcoma, leiomyosarcoma, malignant fibrous histiocytoma, rhabdomyosarcoma, fibrosarcoma, giant cell tumor and anaplastic spindle cell sarcoma [3]. Dedifferentiated chondrosarcoma has a wide variety of imaging features, and it is crucial to identify the imaging features of the dedifferentiated components which underly the chondrosarcoma lesion and determine the growth of the lesion, metastasis, and prognosis of the patients. Radiographs may demonstrate some features of dedifferentiation like aggressive bone destruction, cortical infiltration, a soft tissue mass with no calcification, pathologic fracture and osteoid matrix [3]. Even though some radiologic and magnetic resonance imaging features of dedifferentiated chondrosarcoma have been described [10–13], individual radiologists are still not familiar with the features of this tumor particularly in regard to its multiple malignant mesenchymal components which may have different imaging features. This study investigated the clinical, pathological and imaging features of patients with dedifferentiated chondrosarcoma confirmed by pathology so as to improve the knowledge of this disease for better diagnosis.

## Methods

This study was approved by the ethics committee of the Third Hospital of Hebei Medical University, and all patients had given their signed informed consent to participate. The guidelines outlined in the Declaration of Helsinki were followed in this study. Between 2004 and 2018, patients with dedifferentiated chondrosarcoma confirmed by pathology were included in this study. The clinical, imaging and pathological data were retrieved from the hospital electronic database and analyzed.

## Results

### Clinical data (Table 1)

Twenty-five patients with dedifferentiated chondrosarcoma were identified including fifteen male patients and ten female ones with an age range of 24–74 (median 58, interquartile range 49–65). Three (12%) patients were below the age of 40 years while the other 22 patients were over 40 years accounting for 88%. Ten patients had the tumor at the femur, four at the ilium, two at the humerus, two at the tibia, two at the cotyle, and one at each of the following locations: scapula, sacrum, rib, pubic branch, and calcaneus. Fourteen patients had the tumor at long tabular bones, and eight at the pelvis. Twenty-one patients had local pain and a soft tissue mass while the other four patients had only local pain without a soft tissue mass. Four patients had pathological fractures.

### Imaging presentations

**Fig. 1:**
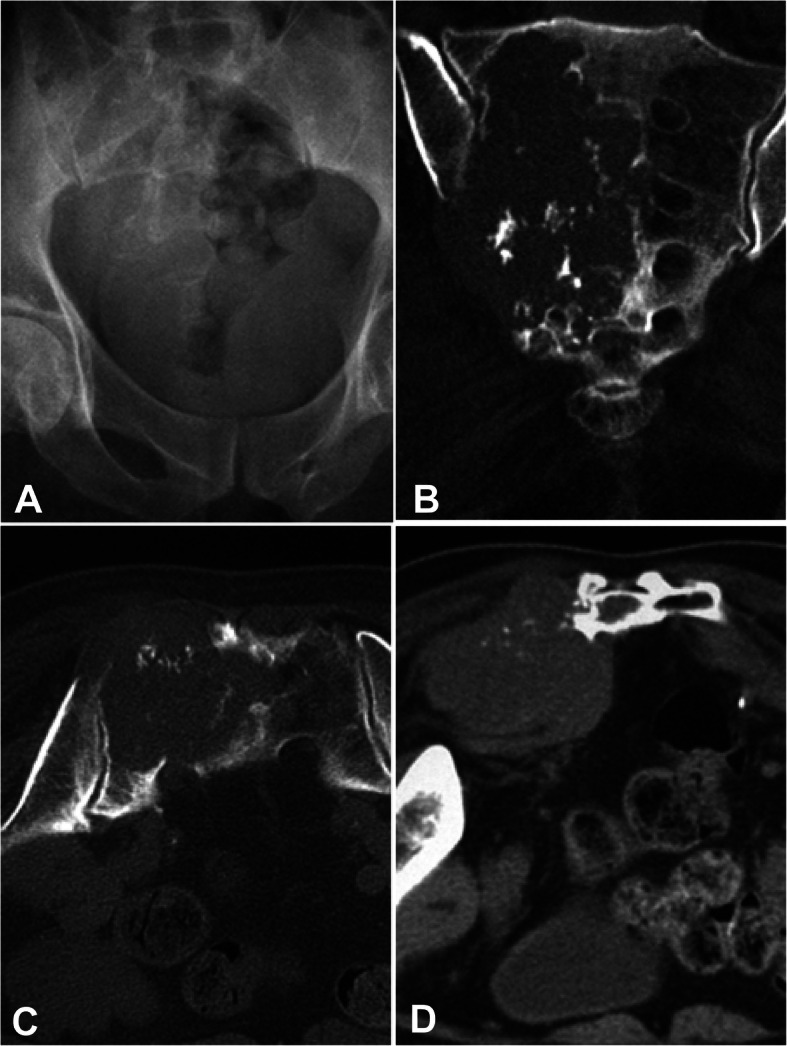
A 70-year-old man had right leg pain for 3 years and was diagnosed with sacral dedifferentiated chondrosarcoma. **a** Plain radiograph showed irregular bone destruction on the right side of sacrum. **b**-**d** Computed tomography revealed irregular bone destruction on the right side of sacrum with punctiform calcification and a soft tissue mass

**Fig. 2:**
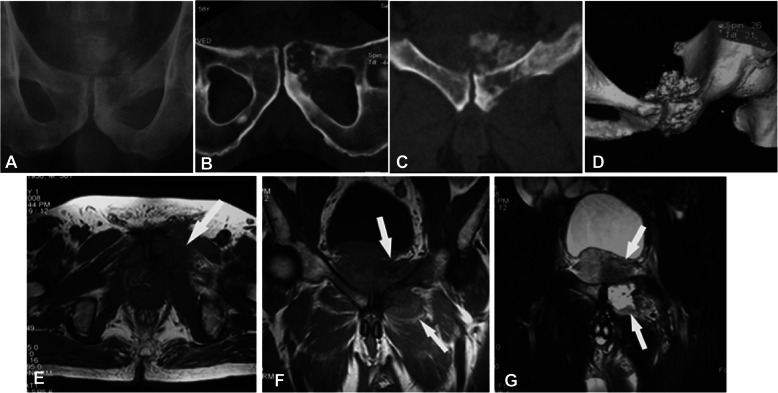
A 58-year-old man had left leg thigh pain for 3 months and was diagnosed with pubic dedifferentiated chondrosarcoma. **a** Plain radiograph demonstrated bone destruction at the pubic symphysis with ill-defined margins and irregular high-density lesion. **b**, **c** Computed tomography (CT) reconstruction displayed the pubic destruction, punctiform calcification and ossification, and a soft tissue mass. **d** Three-dimensional CT reconstruction revealed pubic destruction and a pelvic mass. **e**-**g** Magnetic resonance imaging showed unevenly long T1WI (**e** and **f**) and long T2WI (**g**) signal, with ill-defined margins, calcification, and ossification. The expansile destruction and calcification were typical for chondrosarcoma

**Fig. 3:**
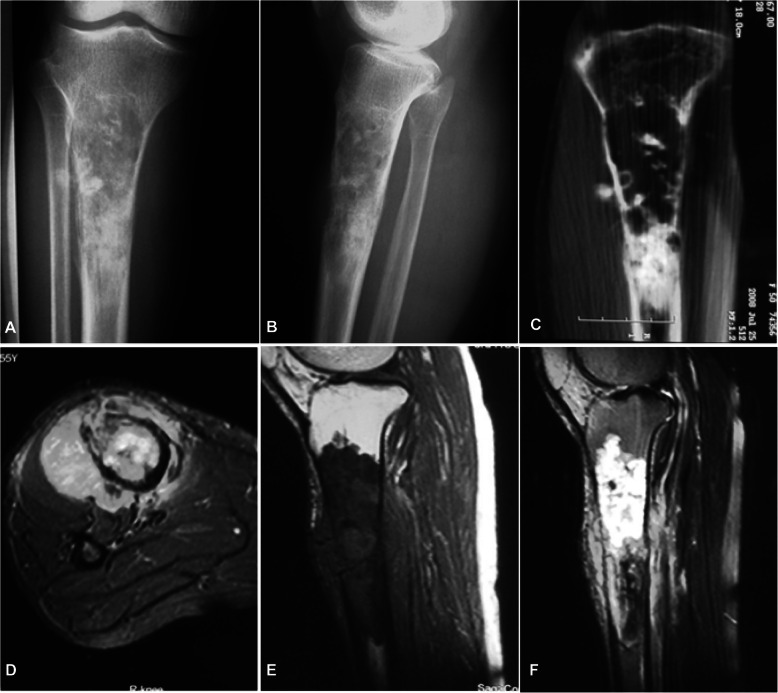
A 55-year-old woman had a mass at the tibia for half a year and was diagnosed with dedifferentiated chondrosarcoma. **a**, **b** Plain radiograph revealed bone destruction at the proximal segment of right tibia with ill-defined margins, irregular circular and patchy calcification, and periosteal reaction. **c** Coronal computed tomography reconstruction showed bone destruction, irregular and circular calcification. **d**-**f**. Magnetic resonance imaging in STIR sequence (**d**), T1WI (**e**) and fat-suppression T2WI (**f**) showed long T1 and long T2 signal with low signal in calcification and ossification. The bone destruction and typical circular calcification of the tumor cartilage are typical for chondrosarcoma

**Fig. 4:**
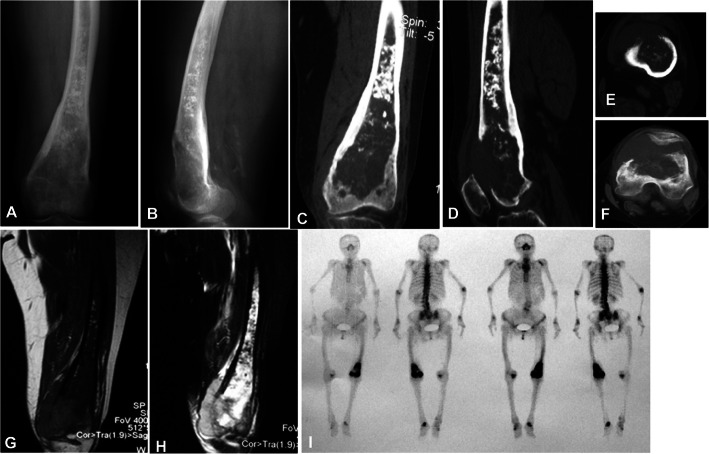
A 75-year-old woman had left leg pain for half a year and swelling of left knee for 5 months and was diagnosed to have dedifferentiated chondrosarcoma. **a**-**f** Plain radiograph (**a** and **b**) and computed tomography (**c**-**f**) displayed calcification within the medullary cavity, osteolytic destruction in the distal end, cortical destruction, and a soft tissue mass. **g**, **h** Magnetic resonance imaging in T1WI (**g**) and T2WI (**h**) showed unevenly long T1 and long T2 signal in the lesion. **i**. ECT scan demonstrated increased radionuclide uptake at the distal femoral segment and assembling of radionuclide at the distal femoral end with some areas of low uptake of radionuclide. This case showed the upper calcified chondroma and lower bone destruction with dedifferentiation

Among fourteen patients with the lesion at the tubular long bones, typical osteolytic destruction appeared with the lesion in two patients having mesh-like or moth-eaten destruction within the medullary cavity, and the other 12 patients had lytic destruction with varying sizes and ill-defined margins (Figs. 1, 2, 3 and 4). The lesion at the cotyle had osteolytic destruction. Twenty-two (88%) patients had calcification or ossification while the other three patients with the tumor at ilium, scapula and tibia had no calcification or ossification. The ossification was located at the edge of the lesion surrounding the osteolytic lesion with ill-defined edge. Calcification was punctiform or circular at the central area of the lesion. Ten (40%) patients had periosteal reaction with the Codman triangle in two cases. Nineteen (76%) had soft tissue masses with well- or ill-defined margins.

### Gross observation of the specimen

The specimen after resection had bone destruction, and the chondrosarcoma components were usually located inside the bone with varying sizes, lobulated morphology, and calcification in some patients. The dedifferentiated sarcoma components were mainly located outside the bone with the maximal size of 10 cm × 15 cm and well-defined margins. Most of the tumors were soft in texture, and tumors with osteosarcoma differentiation were hard in the texture.

### Microscopy

**Fig. 5:**
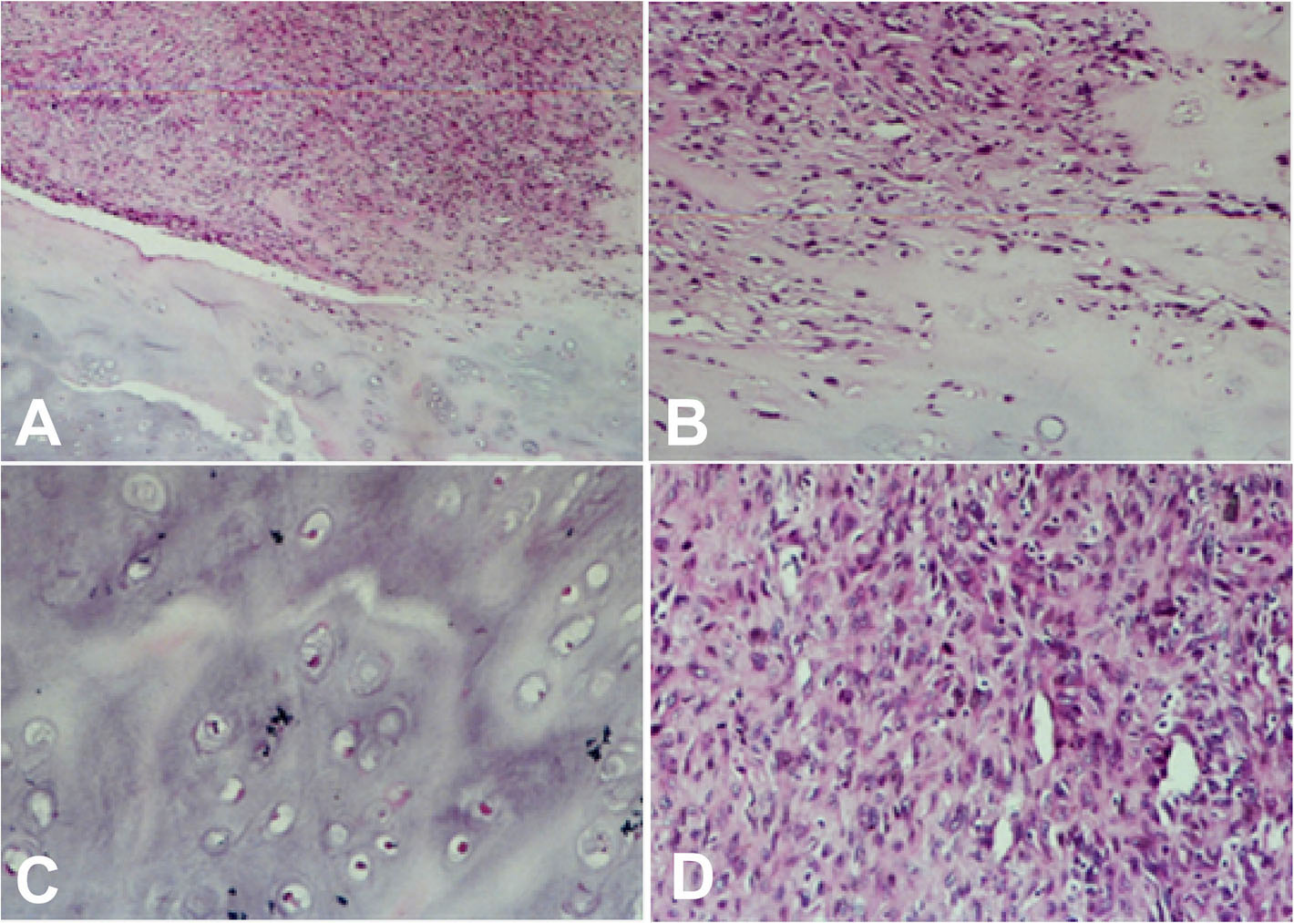
Histopathology of dedifferentiated chondrosarcoma. **a**, **b** The tumor was composed of two components: chondroma and anaplastic sarcoma with clear margins between the two components. **c** The cartilage component was shown in dedifferentiated chondrosarcoma, with only slight cellular atypism. **d** Dedifferentiated component was spindle cell sarcoma

Microscopically, the dedifferentiated tumor had two components: well-differentiated chondrosarcoma and poorly differentiated non-chondral sarcoma (Fig. 5). The well-differentiated chondrosarcoma included well-differentiated chondrocytes, slight atypia, rich hyaline cartilage matrix and accompanied calcification, whereas the poorly differentiated non-chondral sarcoma were composed of spindle or epithelioid cells which were characterized by abundant tumor cells of large volume with obvious atypia, many mitotic images, and pathological mitosis. The boundary between the two components was clear and changed suddenly. Immunohistochemistry showed that vimentin was expressed in 25 cases of dedifferentiated chondrosarcoma, with positive chondrosarcoma S-100 component and positive dedifferentiated chondrosarcoma SATB2. Based on the results of the microscopy and immunohistochemistry, the poorly differentiated non-chondral sarcoma included malignant fibrous histiocytoma in eleven cases, osteosarcoma in ten cases, fibrosarcoma in two, liomyosarcoma in one, and lipoblastoma in the remaining one (Table 1).

### Follow-up

Among 25 patients after imaging examination, amputation was performed in 3 cases, tumor segment resection in 10 cases, and tumor resection plus prosthesis replacement in 12 cases. Followed up from 3 moths to 60 months (mean 15.6), eight patients died with a survival time of 10–23 months (mean 16), and the other 17 patients survived with the survival duration from three to 60 months (mean 15) (Table 1).

**Table 1:** Clinical data of patients

Variables	Data
Age (range, mean, y)	24–74 (55.5)
Sex (F/M)	10/15
Location	
Femur	10
Ilium	4
Humerus	2
Tibia	2
Scapula	1
Sacrum	1
Rib	1
Pubic branch	1
Hip joint	
Cotyle	2
Calcaneus	1
Component	
Malignant histiocytoma	11
Osteosarcoma	10
Fibrosarcoma	2
Liomyosarcoma	1
Lipoblastoma	1
Follow-up time (range, mean, m)	3–60 (15.6)
Follow-up outcome	
Death	8
Survival	17

## Discussion

Dedifferentiated chondrosarcoma occurs mostly in middle aged people between 50 and 60 years of age and frequently in male patients [14]. This tumor was thought to develop through years of evolution of primarily benign chondroid lesion which appears in early youth and possesses enchondroma features [14]. This is because of the specific morphological presentations of the tumor that contains components of the benign cartilaginous hyperplasia immediately adjacent to the well-differentiated chondrosarcoma with low malignancy besides mesenchymal tumor of the high-malignancy grade including malignant fibrous histiocytoma, fibrosarcoma, angiosarcoma and osteosarcoma [14–17]. Dedifferentiated chondrosarcoma usually invades the pelvic bones and other tubular bones like the proximal femur with metaphyseal-diaphyseal propagation of the tumor cells. Clinical presentations may include local edema, pain, limited activities, a soft tissue mass, pathological fracture, and a rapid course of disease measured in weeks rather than in months. Among this group, there were more male than female patients with a median age of 58 years and a large age span. Most of the dedifferentiated chondrosarcomas were located in the long bones and pelvis, accounting for 88% of all cases, which was basically consistent with reports in the literature. Treatment of this tumor comprises combined surgery and chemotherapy primarily based on the type of high-malignancy tumor. Surgical treatment is excision with wide margins or even with amputation [18]. The outcome is reported to be favorable with a radical resection, and local recurrence is associated with inadequate surgical resection margin [8, 18–20]. Nonetheless, due to pathological fractures, lesion permeability, and frequent lung metastases, it is rather difficult to implement wide surgical resection for this tumor [8, 17]. For chemotherapy and radiation, no improvement in the treatment effect and clinical outcomes has been confirmed for these therapies in dedifferentiated chondrosarcoma [3, 11, 15, 16, 18, 20, 21]. Even with optimal therapy, a high rate of local recurrence and death from disease exists with a 5-year survival rate between 7 and 24% [22].

In radiological imaging, the specific characteristics are the juxtaposition of two entities with biphasic nature. Pop-corn type mineralization is the first intraosteal component of the lesion, representing the cartilaginous tumor component (chondrosarcoma). The second component is a lytic lesion with cortical puncture and massive extraosteal proliferation, which corresponds to the presentations of highly malignant tumor. The most common X-ray findings are a calcified tumor within or adjacent to the dominant osteolytic lesion, or CT and MRI show a large non-calcified soft tissue mass connected with a cartilage-like tumor in the bone. CT and MRI are the best imaging modalities to determine the range of lesions. CT can accurately demonstrate fine calcification which cannot be found on X-ray radiography, and MRI can clearly reveal the spread of tumor to extraosseous soft tissues. According to the location of the tumor, dedifferentiated chondrosarcoma can be divided into the central type which is located in the intramedullary region and the peripheral type which is located on the bone surface. The central type of dedifferentiated chondrosarcoma is further divided into the following three types [20]: type I has the imaging presentations similar to those of conventional central chondral sarcoma combined with cortical destruction, soft tissue mass, and pathological fracture, which are considered as the imaging presentations of “defdifferentiation”. Type II presents as progressive malignant transformation on the basis of a benign or low-grade chondrogenic tumor. Type III lacks imaging evidence for chondroma. In our study, all 25 cases were of the central type dedifferentiated chondral sarcoma, with type I in 23 cases and type II in three. Because most of the cases had typical morphological and imaging features of both chondrosarcoma and non-chondrosarcoma, any chondroid lesion with aggressive bone destruction and associated non-mineralized soft tissue mass or with reduced signal intensity on T2 WI magnetic resonance imaging should be suspected of dedifferentiated chondrosarcoma. Because the treatment is mainly determined by the high-malignant component, the morphological and imaging characteristics of both chondrosarcoma and non-chondrosarcoma in the tumor should be paid attention to before surgical operation. Multi-point puncture or biopsy should be performed in different imaging areas so as to obtain the representative sample for pathohistological test which should indicate double components of the tumor: low grade chondrosarcoma and high-grade non-chondrosarcoma. Components of the former may significantly increase the risk of misdiagnosis from benign enchondroma to malignant fibrous histiocytoma. Juxtaposition of the low- and high-grade components is a major diagnostic morphological criterion for differentiation from chondrosarcoma with a fracture and reparation element. The next step is precise determination of the histological type of the high-grade component, which may be one of many tumors like osteosarcoma, malignant fibrous histiocytoma, rhabdomyosarcoma, fibrosarcoma and angiosarcoma. More precisive differentiation may require immunohistochemical phenotype.

Besides the cartilaginous tissue that is morphologically typical and recognizable, mesenchymal tumor element is always present in the dedifferentiated chondrosarcoma lesion. Interpretation of this tumor with multiple components is highly delicate and difficult, especially in cases with pathological fracture. Microscopically, the chondrosarcoma component is usually located in the center of the lesion with frequent calcification inside, whereas non-cartilaginous components are usually located outside the bone with high malignancy and dedifferentiation. The two components had clear demarcation.

Identifying some prognostic factors for dedifferentiated chondrosarcoma is very important to both the patients and doctors due to limited options of treatment, and these prognostic factors may help doctors making good decisions in treatment with future perspective. After studying the radiological characteristics, prognostic factors, and survival, Liu et al. found that axial bone site, lung metastases at diagnosis, incorrect diagnosis prior to surgery, inadequate surgical margin, and pathological fractures were associated with a poorer outcome [8]. Histological subtype of dedifferentiation may affect the survival rate, but the grade of cartilaginous component and local recurrence were not related to the survival. No definitive effect of pre- or postoperative chemotherapy was confirmed on improving survival. Pelvic location, increased age, size (tumor lesion greater than 8 cm), and pathological fracture have also been found or confirmed to be prognostic factors [18, 22, 23]. After investigating C-reactive protein in predicting overall survival of patients with dedifferentiated chondrosarcoma, Nemecek et al. [24] found that elevated C-reactive protein was significantly associated with worse overall survival and was an independent prognostic factor for dedifferentiated chondrosarcoma.

Some limitations may exist in the current study including retrospective nature, a small cohort of patients, single center study and single ethnicity of Chinese people enrolled. Moreover, gene mapping analysis was not performed, either. All these factors may affect the interpretation of the outcome of this study. Future studies will have to address these issues for a better outcome.

In conclusion, dedifferentiated chondrosarcoma is a fatal disease with multiple components, and most of the cases have dual morphological and imaging features of chondrosarcoma and non-chondrosarcoma. The imaging presentations are primarily of common central chondrosarcoma, combined with cortical destruction, soft tissue mass, and pathological fractures, or non-cartilaginous mass combined with osteochondroid lesions. Familiarization with the clinical, pathologic, and imaging characteristics of this tumor helps reaching a correct diagnosis.

## Data Availability

All data and materials are available from the corresponding author on reasonable requirements.
